# Targeted Glioma Therapy—Clinical Trials and Future Directions

**DOI:** 10.3390/pharmaceutics16010100

**Published:** 2024-01-11

**Authors:** Aleksandr Shikalov, Igor Koman, Natalya M. Kogan

**Affiliations:** Department of Molecular Biology, Institute of Personalized and Translational Medicine, Ariel University, Ariel 40700, Israel; aleksandrs@ariel.ac.il (A.S.); igorko@ariel.ac.il (I.K.)

**Keywords:** targeted therapy, anticancer, glioma, brain tumors

## Abstract

Glioblastoma multiforme (GBM) is the most common type of glioma, with a median survival of 14.6 months post-diagnosis. Understanding the molecular profile of such tumors allowed the development of specific targeted therapies toward GBM, with a major role attributed to tyrosine kinase receptor inhibitors and immune checkpoint inhibitors. Targeted therapeutics are drugs that work by specific binding to GBM-specific or overexpressed markers on the tumor cellular surface and therefore contain a recognition moiety linked to a cytotoxic agent, which produces an antiproliferative effect. In this review, we have summarized the available information on the targeted therapeutics used in clinical trials of GBM and summarized current obstacles and advances in targeted therapy concerning specific targets present in GBM tumor cells, outlined efficacy endpoints for major classes of investigational drugs, and discussed promising strategies towards an increase in drug efficacy in GBM.

## 1. Introduction

Glioma is the term referring to brain tumors that arise from glial or precursor cells. This group of oncological conditions includes astrocytoma, oligodendroglioma, ependymoma, oligoastrocytoma, and a few rare histologies. In medical practice, gliomas account for nearly 25% of all primary brain tumors [1]. According to the most recent WHO guideline for the classification of CNS tumors, molecular, genetic, and epigenetic markers are used [2]; however, it is also possible to subgroup gliomas into a grading scale: low-grade gliomas (LGG, WHO grades 1–2) and high-grade gliomas (HGG, WHO grades 3–4).

The most common glioma is glioblastoma (GBM, WHO grade 4 astrocytoma), accounting for 14.3% of all primary brain and central nervous system neoplasms and 49.1% of all malignant brain tumors [1], with a poor prognosis for patients: the 5-year survival rate is at 6.8% [1] and it has a median survival of 14.6 months after diagnosis [3,4]. The patient’s prognosis remains weak despite conventional standard-of-care therapy that includes maximal tumor resection followed by radiotherapy and adjuvant temozolomide (TMZ) administration [3].

To better understand the molecular basis of GBM, extensive genomic [5] and transcriptomic [6] analyses were performed. In 2010, these allowed the classification of glioblastomas into subtypes according to their genetic expression profiles: classical (*EGFR* overexpression, *CDKN2A* deletion, and a lack of *TP53* mutations), mesenchymal (altered *NF1*, *PTEN* mutations, a high activity of *MET* and *CD44*, and *MERTK* genes), proneural (altered *PDGFRA*, mutated *TP53*, point mutations in *IDH1* and the increased expression of *OLIG2*), and neural (expression of neural markers such as *NEFL*, *GABRA1*, *SYT1*, and *SLC12A5*) [5]. Median survival rates of 11.5, 14.7 and 17.0 months for the mesenchymal, classical and proneural subtypes were determined [7].

Even though 2010 Verhaak’s [5] subtyping for GBM pinpointed core transcriptomic and genetic alterations and could still be found in use in clinical practice [8], it was not shown to be efficient for improving treatment for chemotherapy patients [9].

The precise classification of individual tumors is a constant work in progress in order to improve the quality of treatment as well as for a better understanding of tumoral heterogeneity [10,11], with major revisions by the World Health Organization based on advances in the field of cancer studies [2]. Currently, single-cell RNA sequencing for neuronal and tumor stem cells as well as the neuronal microenvironment is used for classification of glioblastomas, which helps to build a full view on the tumor as a system—combining data on alterations within genes, pathways, functions and interplay in transcriptional factors [12]—while initial Verhaak’s expression alteration findings are still used as a core for building research projects and clinical trials [5,8,13].

Data on the precise molecular alterations and interferences with the biochemical pathways within GBM cells create a vast base for potential targeted therapies that can specifically recognize tumor cells. This is a promising field of ongoing research and clinical trials, as the targeted delivery of anti-cancer drugs to GBM could potentially diminish the systemic toxicity of non-targeted therapy and provide a better reach of cytotoxic agents towards malignant cells and therefore improve the prognosis for patients. The need for the development of a better high-grade glioma treatment is very high [14].

In this review, we will outline components of the targeted delivery systems and current approaches to achieve selective delivery of the drugs to gliomas in research and practice, as well as outline the challenges arising from the task of the delivery of components to the brain tissue. The main focus of the review is on therapies for GBM with minor remarks towards other types of gliomas, when necessary.

## 2. Conventional Standard of Care for Glioma Therapy

For glioblastoma patients, surgical resection of the tumor to the maximal feasible extent followed by focal tumor radiotherapy and concomitant temozolomide chemotherapy combined with additional irradiation therapy should be taken as the standard treatment (Stupp treatment) [3]. However, it is known that all glioblastomas will eventually relapse or progress [15]. Notably, there are currently no standardized treatment protocols for recurrent glioblastoma (rGBM) patients. Current approaches for the treatment of recurrent glioblastoma involve non-targeted approaches (for details, refer to the excellent review articles [16,17]) such as surgery, radiotherapy or systemic cytotoxic chemotherapy, while targeted approaches involve the same general tactics (Receptor tyrosine kinases (RTK) inhibition and immune checkpoint inhibition) of treatment that will be discussed further in the review. Further studies dispute the validity of the interpretation of clinical trials’ results due to the usage of historical controls with a lack of controlled experiments in the aforementioned studies and an incomparable study design [18,19].

For low-grade gliomas, the optimal treatment involves immediate surgical resection of the tumor to avoid further cancer progression and allow precise molecular characterization of the tumor [20], which is indeed crucial for the development of a treatment plan for any glioma [2,10,11]. For high-risk cohorts of low-grade-glioma-diagnosed patients (age > 40 years; patients who do not undergo gross total resection surgery [21]), postoperative care consists of 50–54 Gy radiotherapy followed by adjuvant therapy with DNA-alkylating or cytostatic agents. Lomustine, a DNA-alkylating agent, is usually preferred due to its tolerable toxicity and blood-brain barrier (BBB) permeation properties [22]. Taking the disease progression from low- to high-grades and the recurrent nature of gliomas [15], treatment options require constant attention and rapid development. Recently, the main vector of treatment has shifted from radiotherapy to targeted or individualized chemotherapy applications [23,24,25].

There are several FDA-approved drugs and one medical device for glioma management aside from TMZ [26]: lomustine [27], intravenous carmustine [28], carmustine wafer implants [29], bevacizumab [19], and tumor treatment fields [30]. These drugs and devices are mainly approved for the management of recurrent high-grade gliomas with only TMZ, carmustine wafer implants, and tumor treatment fields suitable for application in de novo diagnoses. Except for bevacizumab, FDA-approved drugs for gliomas represent the class of DNA-alkylating agents—their effect is not targeted on tumor cells; therefore, their application is linked with systemic adverse effects.

Vascular endothelial growth factor (VEGF) was shown to be one of the key regulators of malignant angiogenesis in glioblastoma cells, while its inhibition with the help of antisense oligonucleotides correlated positively with reduced tumor growth and, indeed, diminished vasculature formation [31]. This discovery led to the FDA approval of bevacizumab, a monoclonal antibody that targets VEGF-A on the surface of endothelial cells and blocks its interaction with its receptors VEGFR1 and VEGFR2, becoming the first FDA-approved targeting therapy for use in recurrent GBM. Although bevacizumab demonstrated enhanced progression-free survival (PFS), it did not provide a benefit to overall survival (OS). This suggests that solely targeting the VEGFR axis is inadequate for suppressing tumor progression [32,33].

## 3. Delivery of Therapeutic Drugs to the Brain and Associated Difficulties

### 3.1. Blood–Brain Barrier as an Obstacle for Drug Delivery

The blood–brain barrier poses a significant challenge in treating conditions within the central nervous system (CNS) and brain tumors are not an exception. The components of BBB include endothelial cells with tight junctions (specific for CNS), smooth muscle cells, microglial cells, astrocytes and pericytes [34]. Tight junctions in endothelial cells create a nearly impenetrable barrier, restricting the passage of small and lipid-soluble molecules through the paracellular route. Additionally, the absence of fenestrations and an elevated mitochondrial content are characteristics that facilitate the transport of solutes to and from the brain [35,36].

Relevant to drug delivery, the BBB allows the free passage of hydrophilic molecules with a molecular weight of up to 150 Da and hydrophobic molecules with a molecular weight of 400–600 Da [37], while the penetration of large or hydrophilic drugs is limited. In order to travel through the BBB, the drug may utilize receptor-mediated transcytosis mechanism (e.g., with transferrin and insulin receptors [38]). P-glycoprotein-1 (P-gp), which can pump out more than 60% of the marketed drugs, makes it more difficult to penetrate the BBB [39]. Studies have indicated that nearly all (98%) small molecules and all large molecules are unable to cross the blood–brain barrier [40]. Serving as a dynamic interface, the BBB effectively segregates brain tissue from the bloodstream, establishing a biological milieu conducive to neurological activities [41,42,43]. Consequently, many potential chemotherapy agents struggle to traverse the BBB and reach its targets within the brain tissue.

According to a recent review [44], pharmaceutical agents that are active within the brain rely on the hydrophobic–hydrophilic balance; some CNS drugs can cross the BBB through passive diffusion. Notably, the majority of antitumor drugs are subjected to efflux from cells by the action of multidrug-resistance (MDR) proteins like P-gp and MDR-associated proteins (MRPs) which creates a big challenge with the delivery of targeted drugs as often they are administered systemically via intravenous injection.

In the case of brain tumors, the efflux machinery (MRP1 and P-gp) proteins are overexpressed by cancer cells compared to by the surrounding vasculature, which poses a challenge for clinical treatment [45,46]. *P-gp* and *MRP1* are major players in the development of drug resistance as they are upregulated in glioblastoma [47]. Thus, in order to surmount resistance induced by anticancer therapeutics, efflux pumps need to be controlled at the BBB, for which novel drug formulations, drug conjugates and delivery systems could be used [48].

The transcellular route of drug transport through the BBB is favorable for molecules with a low molecular weight (<500 Da) and high lipophilicity [49]. The relationship between the polar surface area and brain permeability is proved to be inverse—drug candidates that have >80 Å of polar surface and a tendency to form multiple H-bonds require higher free energy to penetrate lipophilic membranes of the BBB cells [49,50,51]. However, the predominant usage of less polar or highly lipophilic compounds also has its drawbacks as lipophilic molecules would aggregate bound to plasma proteins, effectively decreasing the amount of available active compound [52]. Alternatively to para- and transcellular routes, drugs could cross the blood–brain barrier via transcytosis, which could be receptor-mediated or induced non-specifically by positively charged molecules, therefore allowing adsorption [53].

For the treatment of high-grade gliomas [54], only a small number of cytotoxic drugs are being used, such as TMZ, which allows about 20% penetration through the BBB at the administered systemic dose [55]. Other well-studied drugs such as paclitaxel (PTX), doxorubicin, and cisplatin, are not included in the standard-of-care treatment for GBM due to their poor BBB permeability, even though preclinical in vitro studies have shown an inspiring decrease in the viability of cancer cells using these drugs [56,57,58]. For a meaningful clinical impact, elevated doses of systemic treatment are necessary at the targeted site. This leads to a heightened occurrence of unwanted adverse effects as a result of the exposure of healthy cells to the drug [59].

A variety of research groups are working on overcoming the BBB in order to deliver pharmacologically active drugs to the brain tumor site, including modification of the active compound through conjugation with BBB-permeating proteins [60,61,62], cell-penetrating peptides [51,63], integrin-targeting polypeptide motifs [64], and BBB-targeting aptamers [65]. While this task could be achievable in experiments in vitro and in vivo [60,61,62,65], clinical trials with such drugs often return limited to insufficient outcomes in terms of clinical efficacy, which leads to abandonment of the drug from further studies [64,66,67,68,69,70]. Many such drugs are administered systemically, either via intravenous infusion or oral application, which is indeed a desirable route of application of targeted drugs as it is safe for patients as well as easy to apply; however, considering the history of disappointing trials’ outcomes, it might be a promising strategy to test drugs through other means of administration such as direct intratumoral delivery [71,72], through the intracerebroventricular path [73,74,75] or by cranial intraarterial infusion [76].

### 3.2. Targeted Drug Delivery to the Brain

#### 3.2.1. Passive Targeting

Passive targeting is a major strategy in drug development to achieve therapeutic effects while using systemic administration, particularly for drugs that need to reach the brain. This strategy takes advantage of the abnormal vessel formation in tumors, resulting in enlarged pore sizes in the vasculature endothelium at the tumor site. As a result, compounds that usually cannot cross the blood–brain barrier can be delivered to the tumor site with a higher probability [77,78]. This condition is known as the Enhanced Permeation and Retention (EPR) effect. Pore cutoff sizes differ drastically in healthy (4–25 nm) and brain tumor blood vessels (380–780 nm). Taking that into account, as well as the fact that lymphatic drainage is also impaired in brain tumors, drug delivery nanosystems that are designed to utilize the EPR effect for reaching the tumor site may be up to several hundred nanometers in diameter and could stay in the targeted area for longer periods of time [79]. The size of the nanostructures is a variable parameter in drug design and could be changed to increase the tumor permeation rate and decrease systemic toxicity [80]. Dual-modality nanosystems, designed for both the diagnostics and therapeutics of gliomas, such as QSC-LP liposomes loaded with quantum dots, supramagnetic iron oxide and cytotoxic peptide cliengitide, have shown promising results in vivo by significantly prolonging overall survival and diminishing tumor size in a xenograft mouse model [81].

Although passive drug targeting is helpful within the task of crossing the BBB, it still faces problems with off-target drug release and associated antitumor efficacy issues. Indeed, utilizing abnormal fenestration within the BBB increases the chance of successful brain delivery; however, in this case, therapeutic formulations often do not have specific molecular targeting markers, which might lead to unwanted cytotoxicity within healthy neural tissues outside the tumor region.

#### 3.2.2. Mechanical Targeting (Local Delivery)

Local application of the therapeutic agents to the tumor site, which we will refer to as ‘mechanical’ targeting, could be achieved via intracerebroventricular injection, intratumoral injection, convection-enhanced delivery, and adding anti-cancer compounds intraoperatively into the post-lesion cavity, such as carmustine wafers [19,70]. In this case, as there is no need to pass the BBB and no application of drugs occurs in the circulation, therefore, the systemic toxic effects are significantly lower than in the case with intravenous application, as well as there being increased therapeutic efficacy of the active compounds as they are delivered locally to the site of the tumor [82,83,84,85]. The local application may involve non-specific drugs that are cytotoxic towards any rapidly dividing cells; however, in this review, we will emphasize the therapies that allow the specific targeting of malignant cells while not affecting healthy tissue around the tumor.

Due to the heterogeneity of malignancies and their individual properties (such as drug application either prior to or after surgical intervention), there are no uniform data regarding the depth of drug infiltration [86]. However, in the studies with polymeric implants for intratumoral application [86] and the micro-syringe chip-guided intratumoral administration of lipid nanoparticles [87], drug penetration was allowed due to tumor perfusion in the areas that are located near the implantation site (mm range, e.g., highest concentrations of doxorubicin in the 2 mm radial area near the implant for the in vivo study of liver cancers [86]).

#### 3.2.3. Active Targeting

Active targeting is a method of drug delivery that utilizes ligands (monoclonal antibodies and their derivatives, peptides, etc.) for tumor-specific recognition. Upon recognition of tumor-specific biomarkers which are represented by overexpressed or mutated surface proteins, the drug promotes its cytotoxic effect either directly or indirectly based on a type of drug system and its corresponding specifications. As an example, contrary to passive drug delivery through the EPR effect described above, targeting BBB-overexpressed glioma markers with corresponding ligands showed successful delivery to the tumor site in a phase I trial of the peptide–drug conjugate GRN1005 [67], as well as nanoparticles in preclinical in vitro [61] and in vivo studies [62,65,88,89].

An outline of actively targeted drug delivery systems and their components is described in the following section.

## 4. Drug Delivery Systems for Active Targeting of Brain Tumors

### 4.1. General Scheme for Drug Delivery Systems That Use Active Targeting

**Figure 1:**
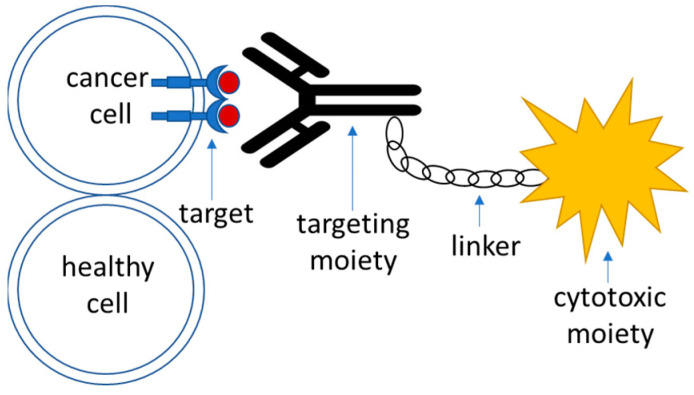
A schematic outline of the components of an active targeting drug delivery system.

Targeted drug delivery systems that utilize active targeting strategy are generally composed of the following parts (Figure 1):

1. Target—this is a moiety that is specific to the cell of interest—in this case, a tumor cell. It is required to be expressed or significantly overrepresented in the tumor over healthy cells; high-level expression is advantageous, although not necessary [90]. It could also represent a molecule within an altered biochemical pathway that is specific to a tumor cell.
2. Recognition moiety—a part of the drug delivery system that recognizes the target specifically and allows address delivery to a specified tumor site with minimal toxicity and off-target effects.
3. Linker—an engineered connective unit that ties the recognition part of the drug delivery system to the payload.
4. Payload—a cytotoxic agent that acts upon delivery to the tumor cells either directly or indirectly through the mediation of the host’s immune response.

### 4.2. Targets

Targeted glioma therapies commonly aim for the recognition/inhibition of the tumor-overexpressed variants of the receptor tyrosine kinase (RTK) family of transmembrane proteins that are responsible for cell growth and proliferation signaling. RTKs represent the most abundant and clinically relevant group of surface targets for GBM drug development [6]. In glioma initiation, several subclasses of RTKs are prone to alterations such as amplification and point mutations [6,91]. Most studied examples include members of epidermal growth factor receptor (EGFR), vascular endothelial growth factor receptor (VEGFR), platelet-derived growth factor receptors (PDGFR), fibroblast growth factor receptors (FGFR), and mesenchymal–epithelial transition factor (MET or c-MET) subfamilies [92].

From a structural point of view, RTKs contain an extracellular domain required for ligand binding, a transmembrane domain, and an intracellular domain with tyrosine residues required for receptor activation upon phosphorylation [93]. The binding of receptor-specific ligands triggers dimerization of the RTK and subsequent autophosphorylation at intracellular tyrosine residues (activation sites). Activated residues act as landing pads for small G-proteins whose recruitment is necessary for downstream signaling through the RAF/MEK/ERK and PI3K/AKT pathways, which control tumor formation, cell survival, proliferation, and invasion [91]. In the context of GBM, alteration in the RTK/PI3K/AKT pathway, which is responsible for constitutive RTK signaling, is considered to be the most elevated core signaling pathway [92]. Alterations (mutations/deletions) in oncosuppressor genes such as *TP53*, *NF1*, and *PTEN* additionally promote RTK activity, which accelerates malignant progression and plays an important role in tumor initiation [92,94].

The other targeting strategy involves the blockade of immune checkpoint inhibitors (e.g., PD-1/PD-L1, CTLA-4, TIM-3, and LAG-3; see Section 5.2) to modulate the immune response towards tumor cells and increase the probability of tumor cell death. The general approach towards targeting immune checkpoint receptors is through the administration of monoclonal antibodies that reactivate the normal lymphocytic response to immunoevasion by cancer cells [95] via T-cellular immunity [96,97]; therefore, immune checkpoint inhibitors act indirectly on the tumor cells by modifying immune cells in the tumor microenvironment. Therefore, using immune checkpoint inhibitors could serve as a potential treatment line for patients with brain tumors [96].

### 4.3. Recognition Moiety

Recognition moieties in the development of targeted drug delivery systems are represented by several classes of molecules. As mentioned before, the recognition particle of the drug should be tumor-specific, with minimal off-target effects as well as no reactivity with healthy tissues [98]. Recognition moieties utilized in drug delivery systems include

- Monoclonal antibodies and their derivatives (such as antibody fragments (Fabs), single chain variable fragments (scFvs), and bispecific antibodies) [99]. The most frequently used format for the development of antibody–drug conjugates is IgG1 immunoglobulin, as it is both easy to manufacture as well as the fact that it shows a strong cytotoxic effect [100]. To promote the cell-killing effect, engineered antibodies could work as both internalizing moieties that release an active drug within the cell of interest [101] or non-internalizing units that release toxic payload in the extracellular matrix of the tumor site affecting malignant cells [102,103,104,105,106]. Monoclonal antibodies could also promote an antiproliferative effect on cancer cells via the receptor blockade, which leads to the depletion of signaling and subsequent cell death [91,107], combining recognition and cytotoxic function in one molecular unit.
- Peptides as recognition moieties in the targeted drugs are presented as either cell-targeting or cell-penetrating units [108]. While the first group’s mechanism of action is similar to that of antibody–drug conjugates, the latter’s is not yet fully understood [95,96]. The uptake and delivery of cytotoxic drugs with the help of peptides as a recognition particle have been shown in preclinical [88] and clinical research [67].
- Small molecules as inhibitors of intrinsic pathways are often used as therapeutic agents in glioma treatment [109,110], as the dysregulation of such pathways (e.g., signaling pathways involving RTKs, PI3K, p53, etc.) has a major role in GBM development [6]. Preclinical data on efficacy and targeted distribution are reviewed elsewhere in detail [110]; however, clinical trials of such compounds often result in disappointing efficacy in the treatment of GBM (see below).
- Aptamers could also be used as a recognition unit of a targeted drug delivery system. Aptamers are short nucleic acid sequences that could be selected based on their affinity towards tumor cells via the systematic evolution of ligands by an exponential enrichment (SELEX) process [111]. It was shown through in vitro [112] and in vivo [113] studies that the PDGFRβ-specific aptamer Gint4.1 can promote cytotoxicity and the delivery of other pharmaceutically active compounds within GBM models.

### 4.4. Linker

In the scope of this review, we will consider any means of connection to the recognition moiety and a cytotoxic agent as a linker for convenience.

For antibody- and peptide-conjugated drugs, linkers could be classified into cleavable and non-cleavable subgroups.

Cleavable linkers in conjugated drugs are developed for the cytotoxic drugs to be released upon cellular uptake of the drug conjugate, which reduces off-target drug release with associated unwanted toxicity in healthy tissues [114,115]. In general, there are three main triggers for linker cleavage and drug release for conjugated drugs: acid-cleavable linkers undergo degradation and drug release due to the usage of the difference between pH levels in plasma (approx. pH 7.4) and endosomal/lysosomal compartments of the tumor cells [116,117] that could be as low as pH 4.5–4.7 [118]; enzyme-cleavable linkers are being cleaved upon internalization in the endosomal or lysosomal compartment of the cell based on the linker structure, with help of intracellular cathepsin B and beta-glucuronidase enzymes [119,120,121,122], and redox-sensitive disulphide linkers undergo reductive cleavage based on the difference in the number of reducing equivalents in the form of glutathione between the plasma (2–20 μM/ L) and cytoplasm (0.5–10 mM) [123].

Non-cleavable linkers in the conjugated drugs require retention of the drug activity upon degradation of the recognition moiety within the endosomal compartment of the cells while the linker or its part stays intact. The advantage of using such types of drugs is associated with a decreased chance of off-target effects, as the drug is more likely to transform to its active form only upon internalization to the cells of interest [124,125,126].

Additionally, the delivery system used in nanoformulations targeted against pathological cells could also be considered as a linker that connects the cytotoxic payload to the recognition part of the drug. Systems that use such kind of linkers in the investigation of brain tumors include lipid and polymeric nanoparticles [127,128], liposomes [129], micelles [130] and metal-based nanoparticles [131,132].

### 4.5. Payload (Cytotoxic Agent)

Cytotoxic payloads that are commonly used in the arsenal of targeted therapeutics include

- Conventional chemotherapeutics such as doxorubicin [133,134] or paclitaxel [67,135,136];
- Microtubule assembly inhibitors: auristatins that inhibit tubulin polymerase and promote cell cycle arrest [137] such as monomethyl auristatin E (MMAE) [138] and monomethyl auristatin F (MMAF) [139], or maytansines and their derivatives that bind to tubulin and therefore interfere with the assembly of microtubules [140]. Common examples include mertansine (DM1) [141,142,143] or ravtansine (DM4) [144,145] as parts of the cytotoxic agents in targeted therapeutic drugs.
- Bacterial toxins—toxic compounds produced by Pseudomonas aeruginosa (Pseudomonas Exotoxin A) and Corynebacterium diphtheria (Diphtheria toxin) are the most commonly used toxins of bacterial origin that are utilized for cancer therapies [146]. Both toxins act upon binding and irreversibly modify the eukaryotic EF2 elongation factor, which results in impaired protein synthesis and cell death [147,148]. In the treatment of GBM, a targeted drug D2C7-IT, comprising of the EGFR-targeted recognition moiety linked to a recombinant pseudomonas exotoxin A, showed promising results in preclinical studies [149] and now patients are being recruited for the evaluation of the drug’s safety and efficacy in Phase I and II clinical trials NCT04547777 and NCT05734560;
- Radioligands fused with peptides or antibodies with tumor specificity are used as a cytotoxic moiety for the targeted delivery of drugs to the tumor sites [71,72,150,151], which allows tumor-specific distribution of the radioactivity as compared to standard beam irradiation in the standard-of-care Stupp protocol [3].
- Photodynamic therapy (PDT) is a therapeutic approach that requires the incorporation of non-toxic and inactive photosensitizer molecules into the cells of interest with subsequent light irradiation, in which the therapeutic molecules become activated. Upon irradiation, photosensitizer molecules transfer light energy and excitate molecular oxygen present in the surrounding tissues to a triplet or singlet state. In the triplet state, oxygen is capable of the generation of reactive oxygen species that react with molecules containing double bonds, leading to their damage and subsequent cell death [152]. In glioma studies and treatment, PDT is implemented via placing fiberoptics at the site of the tumor in order to irradiate cells within the brain that have obtained photosensitizer molecules [153,154]. The clinical application of PDT in glioblastoma patients showed promising results as an intraoperative strategy, with a median PFS of 17.1 months and median OS of 23.1 months [155]. PDT showed a significant improvement in patients’ overall survival compared with the Stupp protocol in patients with non-resectable brain tumors [153] and in clinical trials evaluating the efficacy of PDT in patients with newly diagnosed glioblastoma in intra- and postoperative settings [156,157].
- Oncolytic virotherapy is a novel approach for the targeted therapy of neoplasms that utilizes viruses for the elimination of tumor cells. While the exact mechanisms and the nature of tropism for such viruses might still be unknown, the main theories involve either direct killing of the targeted cells or indirect modulation of the host’s immune system response in order to mediate immunogenic cytotoxicity [158,159]. Clinical trials that have tested oncolytic virotherapy showed increased overall survival as compared with historical controls for polio-rhinovirus chimera PVSRIPO [160]; tumor reduction and safety were observed for tumor-replicative adenovirus DNX2401 with a median overall survival at 13 months for patients who received the treatment intratumorally [161]. For residual and recurrent glioblastoma, the use of a triple-mutated herpes simplex virus showed a median overall survival of 20.2 months after initial tumor resection, which is favorable to any other treatment [162]. This result leads to the governmental approval of an oncolytic virus as a therapeutic modality in Japan (clinical trial registry number UMIN000015995) [162].

However, that general scheme for the drug delivery system is not obligatory required to contain all of the outlined parts except the target on the desired delivery site: some targeted therapies could be linkerless [163] and some could trigger anticancer cytotoxicity through recognition of the target, therefore, being an all-in-one recognition and therapeutic agent (antibody binding to an overexpressed cellular marker which blocks cancer immune evasion and/or interferes with the cell surface receptors, the action of which is required for proliferation of the tumor cells [90,91,107,164]) or by blocking the altered biochemical pathway that is only relevant to cancer cells [110,165].

In this review, we describe the therapeutic drugs that utilize the cellular target as a route for delivery of a cytotoxic payload, which goes in accordance with the four-component scheme of composite delivery drug (in this case, cell surface target bound by a recognition moiety of the drug does not affect the functionality of such a cellular target (e.g., cell surface protein)), as well as drugs that target surface receptors in order to alternate their functionality and therefore lead to cytotoxic events.

For convenience and to avoid redundancy, in this review, the targeted therapies will be sorted by targets.

## 5. Strategies in Clinical Approaches towards the Treatment of GBM

### 5.1. Targeting Receptor Tyrosine Kinases

Alterations in expression within GBM could be observed among five families of receptor tyrosine kinases: EGFR, PDGFR, VEGFR, MET and FGFR (Table 1).

In the following subsections, each family of RTKs outlined above will be discussed with the commentaries regarding the current state of clinical advances toward the treatment of GBM, with targeted therapeutics designed to recognize altered RTKs. Information on clinical trials was screened in the available published data in the context of GBM and used as a basis presentation in this review based on available results with regard to patients’ survival endpoints [171]; additionally, it was accessed at clinicaltrials.gov using search terms outlined in each section of the review with filtering for patients older than 18 years and exclusion of trials with unknown status, imaging studies and those that are not specific to gliomas. The time for a clinical trial is defined as the interval between “first posted” and “last update posted” entry dates for completed studies and “first posted” to “estimated study completion date” for active studies. Survival endpoints are outlined in the table within the “Type of intervention (Outcome)” column, the data obtained from publication referenced in the same column or, otherwise, from preliminary results published on the clinicaltrials.gov webpage of the study. This selection criteria applies to all clinical trials’ data throughout the review.

Since the inclusion criteria for clinical trials differ between the studies, it is impossible to uniformly assess the success or failure of the tested compounds (e.g., survival parameters obtained in de novo diagnosed patient cohorts are different from survival data obtained in the studies for patients who have failed in trials for the other lines of treatment). We focused on representing one clinical trial per drug if there is available survival information; however, since GBM is one of the tumors with poor prognosis, we briefly discuss the general lines of clinical investigation in the main text of the review even if it did not lead to a significantly prolonged survival. The majority of completed trials often do not have published results, making it impossible to evaluate the trials’ outcomes; however, they show the vector of interest in certain strategies for specific targeting in gliomas.

#### 5.1.1. EGFR

Epidermal growth factor receptor is a member of the ErbB family of receptors [172]. Downstream signaling pathways for *EGFR* regulate DNA synthesis and since inclusion criteria for clinical trials differ between the studies, it is impossible to uniformly assess the success or failure of the tested compounds (e.g., survival parameters obtained in de novo diagnosed patient cohorts are different from survival data obtained in the studies for patients who have failed in trials for the other lines of treatment). We focused on representing one clinical trial per drug if there is available survival information; however, since GBM is one of the tumors with poor prognosis, we briefly discuss the general lines of clinical investigation in the main text of the review even if they did not lead to significantly prolonged survival. The majority of completed trials often do not have published results, making it impossible to evaluate the trials’ outcomes; however, they show the vector of interest in certain strategies for specific targeting in gliomas. [173].

Glioblastoma somatic genomic landscaping revealed that 57% of GBM cells harbor *EGFR* genetic alterations [6]. *EGFR* amplification and overexpression were identified in 40 and 60% of primary glioblastomas, respectively, often leading to aggressive GBM tumors resistant to treatment, and enhanced proliferation, invasion, and survival [174,175,176,177]. Point mutations and deletions are also observed among GBM patients with around 20% of diagnosed cases [92] harboring a deletion of exons 2–7, resulting in a truncated mutant variant III (*EGFRvIII*) [178].

As of May 2023, 77 clinical trials were listed on clinicaltrials.gov for targeting EGFR in gliomas (search terms “Glioma|EGFR”).

At the moment, only five investigational drugs that are being tested in clinical trials correspond to the full scheme of the system for active EGFR-targeted delivery (Table 2).

Depatuximab–mafodotin, which contains an internalizing EGFR/EGFRvIII-specific antibody linked to the cytotoxic agent monomethyl auristatin F via a non-cleavable maleimidocaproyl (MCC) linker, did not show increased survival rates when tested in combination with conventional radiotherapy and temozolomide in NCT02573324 [182] or as a monotherapy versus conventional treatment in NCT02590263 and NCT02343406 [183,184], as well as it not having an impact on the quality of life of the patients with recurrent glioblastoma [185]; however, preclinical evaluation in vivo showed total tumor regression in mice bearing U87MGde2–7 model tumors, as well as it showing significant tumor growth reduction in patient-derived xenograft models [186].

AMG 595, composed of the maytansinoid DM1 toxin attached to a highly selective anti-EGFRvIII antibody via a non-cleavable linker, showed promising cytotoxic effects in vitro and in vivo [141] and was tested in a phase I trial (NCT01475006) on a group of 32 patients with recurrent glioma, in which 2 patients showed a partial response, 15 showed stable disease and 15 showed progressive disease outcomes [179]. Unfortunately, survival analysis was not an objective of the study and no further studies with AMG 595 are listed on clinicaltrials.gov (https://clinicaltrials.gov/search?cond=Glioma%20%7C%20EGFR&intr=AMG%20595, accessed on 1 January 2024).

The D2C7-IT dual-specific immunotoxin, comprising an EGFR wild-type and mutant-specific (EGFRvIII) monoclonal antibody (mAb) fragment linked to a genetically engineered form of the Pseudomonas exotoxin A via PCR, is currently being investigated in two active clinical trials, NCT04547777 and NCT05734560, with predicted completion in 2025 and 2028, respectively. Preliminary results summarized in a study abstract by [187] show that this drug might be promising for patients diagnosed with malignant glioma.

EGFR(V)-EDV-Dox is a drug in development that is composed of Salmonella typhimurium-derived EnGeneIc O-polysaccharide nanoparticles (minicells) packed with doxorubicin as a cytotoxic agent, which is attached to a bispecific antibody to both a component of the delivery system and EGFR (panitumumab scFv). In the NCT02766699 clinical trial, it showed results of a PFS of 1.6 months and an OS of 9.7 months in monotherapy with dexamethasone, paracetamol, and promethazine pretreatment [180].

C225-ILs-Dox is another example of a targeted glioma drug that contains a panitumumab anti-EGFR antibody as a recognition moiety linked to doxorubicin via maleimide groups at the DSPE-PEG termini. In a pharmacokinetic NCT03603379 study, this therapeutic agent, which was tested in a group of nine patients, showed a median PFS of 1.5 months and median OS of 8 months; however, it is important to note that the enrolment group was small as well as the fact that each patient was tested with different dosing amounts of C225-ILs-Dox as well as having different prior treatments; therefore, median survival data should not be considered reliable [181].

The main approaches in targeting EGFR in gliomas lie within the use of monoclonal antibodies as therapeutic drugs themselves and through the administration of small molecule inhibitors of the tyrosine kinase receptor family (tyrosine kinase inhibitors, TKIs) (Table 3). Both groups of therapies act by binding to EGFR overexpressed on tumor cells and blocking the subsequent signaling required for cell growth.

The first proposed antibody for EGFR targeting was the chimeric human–murine monoclonal antibody Cetuximab. Unfortunately, phase II clinical trials for this drug did not show therapeutic benefit for patients with relapsed GBM, both as monotherapy, showing median PFS of 1.9 months and OS of 5.06 months [69], and in combination with bevacizumab (a monoclonal antibody targeting neoplastic angiogenesis) and irinotecan (DNA synthesis blocker), showing median PFS of 16 weeks and OS of 30 weeks [190].

The first fully human monoclonal antibody targeting EGFR, panitumumab, did not show promising results in a phase II clinical trial combined with the administration of irinotecan; the study was terminated due to not showing efficacy (NCT01017653).

Nimotuzumab, a humanized anti-EGFR antibody, was tested in a phase III clinical trial (NCT00753246) comparing its efficacy in a treatment combined with a standard Stupp protocol (tumor resection followed by radiotherapy and temozolomide), in which no significant prolongation of the OS and PFS was observed for patients with both recurrent and primary glioblastomas [201]. Unfortunately, these results were not in agreement with promising in vivo studies showing that nimotuzumab enhances tumor growth suppression by temozolomide [202], as well as with previous phase II clinical studies showing prolonged survival for patients undergoing radiotherapy with concomitant nimotuzumab [203].

Sym004—a combination of two recombinant monoclonal antibodies targeting different non-overlapping epitopes of EGFR—futuximab and modotuximab—was tested in the NCT02540161 phase II study for recurrent disease. Preliminary results, available at clinicaltrials.gov, showed unsatisfactory results, with progression-free survival among patient cohorts not exceeding 9.95 months for non-bevacizumab failures and 5.51 months for patients who failed treatment with bevacizumab.

The monoclonal antibody GC1118, targeting EGFR, which showed better affinity to EGFR than cetuximab and panitumumab in a preclinical setting [204], is being investigated as a monotherapeutic agent in a clinical trial (NCT03618667).

Bispecific antibodies targeting the mutant variant III of EGFR as well as the T-cell-specific marker CD3 are gaining interest in the clinical setting as they could potentially direct T-cells to lyse GBM tumor cells bearing an EGFRvIII-mutated receptor. Based on preclinical data gathered on cell lines in vitro and on mice xenograft models, as well as toxicity data obtained from cynomolgus monkey experiments, bispecific targeting of EGFRvIII simultaneously with the CD3 T-cell-specific biomarker shows a promising field for treatment of GBM [188], with such therapies as AMG 596 and RO7428731 currently being investigated in phase I clinical trials (NCT03296696 and NCT05187624, respectively).

Clinically tested EGFR-specific small molecule inhibitors with available results include erlotinib and gefitinib. Erlotinib is a reversible inhibitor of EGFR tyrosine kinase activity. The monotherapy administration of erlotinib in clinical trials returns various survival endpoints, possibly due to different inclusion criteria in each study (see Table 3); however, the general trend is in the fact that mostly the OS and PFS endpoints of such studies do not reach levels of the standard-of-care therapy. Combinatorial studies with erlotinib showed no improvement in OS for patients coadministered with bevacizumab [198] and multitarget tyrosine kinase inhibitor sorafenib that targets VEGFR, PDGFR, c-Kit and RET [196].

Gefitinib is a reversible and specific inhibitor of EGFR tyrosine kinase activity. Monotherapy in GBM showed a median OS for patients only at a level of 8.8 months [200]. The clinical trial that tested gefitinib with multitargeted tyrosine kinase inhibitor cediranib versus cediranib plus a placebo showed a trend on prolonged survival rates with patients administered with a combined scheme; however, the study was underpowered statistically, and more investigations should be performed in order to validate that hypothesis [199]. In newly diagnosed GBM patients, combinatorial treatment with radiotherapy and gefitinib did not show beneficial clinical relevance when compared with radiotherapy alone [205], nor is it improved when gefitinib is used as a post-irradiation adjuvant therapy [206].

Novel molecular inhibitors of EGFR are currently being investigated in clinical trials as monotherapy with the EGFR-specific ERAS-801 drug (NCT05222802), as well as in combination with temozolomide vs. monotherapy, for the mutant-specific EGFR inhibitor BDTX-1535 (NCT05256290).

#### 5.1.2. PDGFR

Platelet-derived growth factor receptors are a family of six subunit homo- and heterodimers that form tyrosine kinase receptors, whose physiological function involves the regulation of hematopoiesis, embryogenesis, and glial cell development, and the protection of neuronal tissue [207]. PDGFR alteration and genetic mutations play a driving role in GBM cell proliferation and malignancy, including the dedifferentiation of glial cells and epithelial-to-mesenchymal transition, as well as the effects of intratumoral vessel formation and immunosuppression [208,209].

In GBM patients, amplification and genetic alterations in *PDGFR* are found in 13% of diagnosed cases, overexpression of this RTK is related to poor prognosis [6]. Activation of PDGFR signaling in glioma cells induces malignant vascularization, cell survival, and growth [112,210], and dysregulation of normal PDGFR activity in tumors leads to oncotransformation of healthy glia and GBM growth through autocrine signal transduction [211,212].

As of May 2023, 14 clinical trials are listed on clinicaltrials.gov for targeting PDGFR in gliomas (Selected trials with available information on survival are represented in Table 4, search terms “Glioma|PDGFR”).

In recent clinical trials, the majority of investigational anti-PDGFR agents are small molecule inhibitors (see multitarget kinase inhibitors) and monoclonal antibodies such as MEDI-575 and olaratumab (Table 4) [171].

MEDI-575 is a PDGFRα specific monoclonal antibody that was tested in a Phase II study (NCT01268566) and is a monotherapeutic agent. It showed disappointing clinical outcomes as the median PFS for patients was only at the 1.4 months level with median overall survival at 9.7 months [213].

Olaratumab—a PDGFRα specific monoclonal antibody—was tested as a monotherapeutic agent in a phase II NCT00895180 clinical trial and showed median overall survival at 34.3 weeks level, with 7.5% of patients from the recruitment cohort reaching 6 months progression-free survival endpoint and only 2.5% of the olaratumab treated cohort achieving response from the treatment.

Aside from targeted monoclonal antibodies, several multitargeted tyrosine kinase inhibitors were tested clinically, however, no promising results were seen in the trials involving such drugs with examples of dasatinib, imatinib, and sunitinib [171].

A promising line of investigation within the field of PDGFR targeting in glioblastoma could be targeting PDGFRβ which was shown to be overexpressed in GBM endothelium [154]. Preclinical studies showed that usage of PDGFRβ specific aptamer conjugated to a drug delivery system loaded with PI3K inhibitor daclosiclib might be useful for future clinical trials applications as such delivery system was successful in tumor-specific delivery of the drug in the in vivo mice model while in vitro this system showed successful internalization and cytotoxicity towards glioblastoma U87MG cell line [113].

#### 5.1.3. VEGF/VEGFR

Neoplastic vessel formation is one of the hallmarks of malignant tumor growth and progression, with VEGF receptors and ligands signaling pathways being a potent driver of angiogenesis [214]. Extensive vascularization as a result of angiogenesis is one of the tumor hallmarks, indeed present in glioblastoma with *VEGF* being detected in 64% of diagnosed GBM cases [167] and up to 17% of diagnosed cases showing amplification of the *VEGFR2* gene [168]. Several reports outline that VEGF/VEGFR axis members are strongly upregulated in glioblastoma with a direct correlation to the level of malignancy of the tumor and therefore *VEGF/VEGFR* amplification could serve as a marker of a poor prognosis [215,216]. VEGF/VEGFR signaling in the context of tumor plays a role in tumor growth [217] and proliferation of the cancer stem cells [218] as well as it is responsible for tumor immunoevasive microenvironment [219].

As of May 2023, 73 clinical trials are listed on clinicaltrials.gov for targeting VEGF or VEGFR in gliomas (search terms “Glioma|VEGF”, “Glioma|VEGFR”). Selected clinical trials are represented in Table 5.

Since 2009, when bevacizumab (VEGF-A specific monoclonal antibody) was approved by the FDA the for treatment of recurrent glioblastoma [223], it has become a treatment of reference for combinatorial clinical trials within recurrent GBM. Two major lines of clinical investigation exist in targeting VEGF/VEGFR axis, similar to PDGFR treatment strategies—usage of monoclonal antibodies and small molecule inhibitors, which are mostly multitargeted TKIs and therefore described in the later section of the review.

The combination of bevacizumab with irinotecan (DNA topoisomerase-I inhibitor) showed a promising trend in increased progression-free survival for the patients [223,228] and was a basis for combinatorial trials of bevacizumab with standard-of-care therapy, radiotherapy, and conventional chemotherapeutics (outlined in the table below). Surprisingly, in the majority of the studies that involve bevacizumab as a monotherapy or a part of a combinatorial treatment, only progression-free survival is increased significantly while no therapeutic benefit is observed for the overall survival of the patients [32,220,222,225,226].

CT-322, an investigational peptide drug that is comprised of a modified extracellular domain of fibronectin 10th type 3 with a specificity towards VEGFR-2 was tested in a phase II clinical trial NCT00562419 as a monotherapy or in combination with irinotecan.

CT-322 acts as a high-affinity blocker of VEGFR-2 preventing its binding with VEGF ligands, most importantly, VEGF-A, therefore blocking VEGF-A induced dimerization of the receptor and subsequent MAPK signaling [229].

While showing adequate tolerability and side effects profile, this compound did not reach the trial’s prespecified efficacy measures and was terminated [227].

#### 5.1.4. c-MET Pathway

Another example of a receptor with tyrosine kinase activity that is altered in GBM cells is c-MET. According to glioblastoma molecular landscaping, 1.6–13.1% of diagnosed cases harbor *c-MET* overexpression, with gains in *MET* found in 47% of primary and 44% of recurrent GBM cases [6,169] and up to 4% of cases present amplification of *MET* [92,170]. Alterations in *MET* and its ligand hepatocyte growth factor *(HGF)* are associated with tumor growth, migration, invasion, and development of therapy resistance [230,231,232,233,234].

Since it was shown that inhibition of angiogenesis through blockade of the VEGF/VEGFR axis stimulates c-MET activation and leads to an invasive form of glioblastoma [235], it is important to target the c-MET pathway to effectively treat tumors. In clinical trials, c-MET pathway is targeted with monoclonal antibodies with specificity towards c-MET receptor or its ligand HGF [13,171]. Selected clinical trials are outlined in Table 6.

As of May 2023, seven clinical trials are listed on clinicaltrials.gov for targeting c-MET in gliomas (search terms “Glioma|c-MET”).

Onartuzumab, a monoclonal antibody targeting c-MET, was investigated in a clinical study NCT01632228 and showed a trend towards reducing tumor growth, however, no significant increase in median overall survival and progression-free survival were observed in patients undergoing combinatorial therapy with onartuzumab and bevacizumab as compared to bevacizumab plus placebo [236].

Rilotumumab, an anti-HGF monoclonal antibody, was tested in a NCT01113398 clinical study in combination with bevacizumab pretreatment. Unfortunately, this investigational drug did not show a single-agent antitumor activity in the cohort of patients enrolled for the study [237].

#### 5.1.5. FGFR

FGFR family of receptor tyrosine kinases is comprised of four transmembrane receptors named FGFR1–4 [238]. Upon activation of FGFRs, downstream signaling includes the ERK/MAPK pathway, which in turn is responsible for the regulation of cell survival, proliferation, angiogenesis, development, and differentiation [239,240,241,242]. Even though only 3.2% of glioblastoma patients harbor amplification or point mutation in *FGFR* genes [6], it was shown that GBM cells could evade EGFR and MET inhibition through FGFR-mediated bypass, therefore pointing FGFR inhibitors as possible potentiators of EGFR or MET-targeted therapies [243].

As of May 2023, six clinical trials are listed on clinicaltrials.gov for targeting FGFR in gliomas (A clinical trial with preliminary results is represented in Table 7, search terms “Glioma|FGFR”).

Currently, the research in the field of targeted FGFR inhibitors is mostly preclinical with limited information available for clinical trials in the context of GBM. One clinical trial evaluated the effect of pan-FGFR inhibitor infigratinib in patients with recurrent glioblastoma (NCT01975701) after this drug showed potent inhibition of FGFRs and cell growth on a panel of cancer cell lines in vitro [244], resulting in progression-free survival of 1.7 months and median overall survival of 6.7 months, Unfortunately, infigratinib as a therapeutic compound was outlicenced since then and no further trials and investigations were performed [171].

In preclinical studies, orally bioavailable inhibitor of FGFRs1–3 AZD4547 showed inhibition of tumor growth in vivo in mice xenograft models with KMS11, KG1a, and CaLu-6 cancer cell lines [245] and later translated into a phase I/II clinical trial NCT02824133 for patients with recurrent malignant glioma. However, no published results regarding efficacy endpoints are available up to date.

Erdafitinib (JNJ-42756493) is another example of a preclinical investigational drug that showed inhibitory activity in vitro and in vivo in cell lines and tumor models bearing FGFR alterations [246]. The first-in-human phase I case study for erdafitinib showed clinical improvement for two GBM patients who received this drug validating the use of JNJ-42756493 as a targeted therapy for the inhibition of FGFR [247].

#### 5.1.6. Multikinase Inhibitors

In many cases, with tyrosine kinase receptor-targeted treatments, small-molecule inhibitors, which target multiple tyrosine kinases, are used in clinical trials. Outlined information on those drugs can be found in the table below (Table 8), representing multitargeted tyrosine kinase inhibitor studies that had information on the survival endpoints as a part of efficacy evaluation.

Imatinib is the first multikinase inhibitor whose targets are PDGFRα/β, BCR-Abl, and c-Kit. The initial trial of this drug in combination with hydroxyurea did not show an increase in survival for patients [248]; however, it was tested in trials after that, returning unsatisfactory or clinically insignificant results both in combination with hydroxyurea [249] and as monotherapy [250].

Dasatinib, an inhibitor of PDGFRβ, EPHA2, BCR-Abl, c-Kit, and SRC, was tested in clinical trials on patients with recurrent glioblastoma as a monotherapeutic agent and in combination with chemotherapy, bevacizumab or the conventional standard-of-care Stupp protocol. The monotherapeutic application of dasatinib failed in a clinical trial NCT00423735 as the study did not pass the efficacy preset and was terminated [252]. The combination of dasatinib with lomustine proved to be ineffective due to the efficacy endpoints not reaching the measures of historical controls [253]. Two completed clinical trials that evaluated the coadministration of dasatinib with bevacizumab, versus bevacizumab alone or combined with the Stupp protocol, versus the Stupp protocol alone, did not reach statistical significance for the compared groups and, therefore, the results obtained were unreliable (NCT00869401) [251].

Tandutinib, an inhibitor of PDGFRβ, FLT3, and c-Kit, was tested in Phase II studies as a monotherapeutic agent or in combination with bevacizumab in patients with recurrent GBMs. The results from both showed a lack of efficacy of the drug or its combination and were closed [254,255].

Sunitinib, an inhibitor of PDGFRα/β, c-Kit, VEGFR1/2/3, FLT3, and RET, also showed upsetting results in clinical trials with no significant antitumor activity for patients with recurrent glioblastomas [256,258], while in one study a sub-cohort of patients with high c-Kit expression undergoing monotherapy with sunitinib showed median PFS of 16.0 months and median OS of 46.9 months, with the rest of the tested cohorts showing PFS and OS endpoints at 2.2 and 9.4 months, respectively [257]. This observation might prove valuable that treatment with sunitinib needs to be fine-tuned to a specific cohort of glioma patients.

Ponatinib, a multikinase inhibitor that targets BCR-Abl, PDGFRα, VEGFR2, FGFR1, and Src [260] but also RET, c-Kit, and FLT1, is under investigation in an NCT02478164 clinical trial as a monotherapy for patients with bevacizumab-refractory GBM. Preliminary results show a median OS of 98 days and PFS of 28 days, with minimal activity of the investigational drug in the selected group of patients [259].

Cediranib is a multikinase inhibitor with activity specific to VEGFR, c-Kit, and PDGFR [261]. In a combinatorial clinical trial that compared cediranib + Stupp treatment versus Stupp treatment alone, a statistically significant increase in progression-free survival was observed for a combinatorial administration; however, no increase in overall survival was observed (NCT01062425).

### 5.2. Targeting Immune Checkpoints

To avoid a normal immunogenic reaction, cancer cells have developed several mechanisms to escape surveillance and immune destruction [262]. One of the ways to do so by tumor cells is through “immune checkpoints”—cellular receptors that are expressed by the immune cells in the tumor microenvironment, they could be used as targets for cancer therapy with immune checkpoint inhibitors [263]. Unlike conventional chemotherapy that acts directly on the malignant cells, immune checkpoint inhibitors are working indirectly by modulating immune response within the tumor environment by restoring the ability of the organism to adequately respond to diseased cells via T-cellular immunity [96,97].

The general line of approach towards targeting immune checkpoint receptors is through the administration of monoclonal antibodies that disrupt the negative regulation loop for T-lymphocytes and therefore reactivate lymphocytic response to diminishing immunoevasion for cancer cells [95]. In the glioma context, immune checkpoint receptors that pose as targets for monoclonal antibody blockade include programmed cell death protein 1 (PD-1) and programmed cell death ligand 1 (PD-L1), cytotoxic T-lymphocyte associated protein 4 (CTLA-4), TIM-3 and LAG-3 [96,264].

#### 5.2.1. PD-1/PD-L1

Clinical trials that have investigated the effect of blockade of PD-1 on host T-lymphocytes with the PD-L1 ligand through the use of monoclonal antibodies in glioblastoma were performed for a variety of developed investigational drugs.

As of May 2023, 80 clinical trials are listed on clinicaltrials.gov for targeting PD-1 or PD-L1 in gliomas (search terms “Glioma|PD-1”, “Glioma|PD-L1”; selected clinical trials are represented in Table 9).

Pembrolizumab, an anti-PD-1 monoclonal antibody, was tested in the NCT02337686 phase II trial for patients with recurrent glioblastoma. The investigation tested the effect of the adjuvant or neoadjuvant application of a checkpoint inhibitor for patients undergoing tumor resection. When compared between adjuvant and neoadjuvant cohorts, the addition of pembrolizumab significantly prolongs overall survival (13.7 vs. 7.5 months for neoadjuvant vs. adjuvant therapy) as well as a progression-free survival rate (3.3 vs. 2.4 months for neoadjuvant vs. adjuvant application).

A NCT02337491 clinical trial that showed the monotherapeutic application of pembrolizumab is inefficient compared to combination therapy with anti-angiogenic monoclonal antibody bevacizumab. The result of the clinical trial suggested that while there was no statistically significant prolongation of overall survival for both treatments, the combination of anti-PD-1 therapy with anti-VEGF agent bevacizumab significantly prolongs event-free survival in patients with recurrent glioblastoma (1.4 vs. 4.1 months, for pembrolizumab monotherapy vs. combination with bevacizumab) [266].

In the monotherapy cohort for patients treated with pembrolizumab (NCT02054806), a durable anti-tumor trend was observed with a median overall survival rate of 13.1 months and median PFS rate of 2.8 months, suggesting that single-agent application of this drug might be beneficial for a certain subpopulation of GBM patients. However, as the authors suggest, the increase in OS might be unclear as the baseline characteristic of patient records used as a historical cohort in that study might be non-uniformed and, therefore, this result should be taken with caution [267].

Another example of an anti-PD-1 monoclonal antibody investigated in the clinical setting for GBM patients is nivolumab. It was tested in patients with recurrent glioblastoma in a phase III NCT02017717 [268] trial as a monotherapy vs. bevacizumab monotherapy, as well as in two phases III trials in newly diagnosed glioblastoma patients with methylated ([269], NCT02667587) and unmethylated *MGMT* promoter ([270], NCT02617589). *MGMT*-related studies compared combinatorial treatment with nivolumab and concomitant radiotherapy with temozolomide compared to RT/TMZ alone. Unfortunately, all three studies did not improve survival rates for the patients receiving an investigational drug implying that nivolumab as a monotherapy or in combination with Stupp protocol treatment is ineffective towards GBM.

Additionally, the anti-PD-L1 monoclonal antibody durvalumab, targeting PD-L1 ligand expressed on tumor cells, was also investigated in a phase II study on patients with newly diagnosed and recurrent glioblastoma ([271], NCT02336165); however, no patients achieved efficacy endpoints; therefore, proving durvalumab ineffective in GBM patients with bevacizumab-naïve or refractory background [271].

#### 5.2.2. CTLA-4

Cytotoxic T-lymphocyte associated protein 4 (CTLA-4) is an immune checkpoint receptor that acts upon binding with ligands CD80 and CD86 present on antigen-presenting cells leading to inhibition of T-lymphocyte stimulatory signaling [272]. CTLA-4 expressing T-lymphocytes are usually present in the lymph nodes unlike PD-1-expressing cells in the tumor microenvironment [273].

As of May 2023, 12 clinical trials are listed on clinicaltrials.gov for targeting CTLA-4 in gliomas (A clinical trial with preliminary results is represented in Table 10, search terms “Glioma|CTLA-4”).

Tremelimumab, an anti-CTLA-4 monoclonal antibody, was recently tested in a phase II NCT02794883 clinical trial as a monotherapeutic agent or combined with anti-PD-L1 monoclonal antibody durvalumab. A combination of these drugs showed contradictory results as compared with durvalumab alone; however, the combination of durvalumab with tremelimumab might be beneficial for increasing the PFS rate of the patients.

Preclinical research investigating CTLA-4 immune checkpoint blockade in mice bearing intracranial GL261 syngeneic glioblastoma showed that treatment with anti-CTLA-4 monoclonal antibody results in a 25% cure rate and prolonged overall survival (27.5 vs. 40 days, non-treated vs. anti-CTLA-4 mAb treated mice); however, coadministration of anti-CTLA-4 and anti-PD-1 monoclonal antibodies results in a 75% cure rate among mice tested with an increase in overall survival from 27.5 to >146 days [274]. This result might be an important hint for planning future clinical studies as it may be beneficial to target CTLA-4 in combination with other immune checkpoints in a single line of treatment for GBM. Preclinical studies implementing multiple immune checkpoint blockers support the idea of coadministration of such drugs: in vivo experiments with triple blockade of CTLA-4, PD-L1, and IDO showed prolonged survival for treated (>90 days) vs. untreated mice (29 days) [275].

For further investigation, CTLA-4 blockade as a monotherapy or in combination with anti-PD-1 treatment is therefore currently being tested in a phase III clinical trial in patients with recurrent GBM (NCT02017717). The results from that study available at the moment show that treatment with anti-PD-1 therapeutics has no effect on overall survival and fails to achieve prolonged progression-free survival if compared to anti-VEGF therapy in recurrent glioblastoma [268]; the data on comparison between anti-PD-1 and anti-CTLA-4 treatment are unpublished and therefore unavailable.

In a phase I clinical trial of NCT03233152 that tested a combination of anti-CTLA-4 monoclonal antibody ipilimumab with a coadministered anti-PD-1 monoclonal antibody nivolumab in patients with recurrent GBM, a positive trend towards prolonged overall survival was observed (median OS = 38 weeks), with no significant change in PFS (11.7 weeks) when compared with historical controls of GBM patients treated with anti-VEGF therapy [276]. At this moment, other clinical trials, whose purpose is to test combinatorial anti-CTLA-4 and anti-PD-1 therapy, are ongoing (NCT04323046 and NCT04606316).

#### 5.2.3. TIM-3

TIM-3 is an immune checkpoint that is widely expressed in glioblastoma and is an important regulator of the inflammatory response related to anti-PD-1 inhibition by therapeutic agents [277,278].

Thus, the combined anti-TIM3 inhibitor and other immunotherapies are getting promising. In preclinical investigation, the combination of anti-TIM3 therapy, anti-PD-1 therapy, and radiotherapy in animal models has reported promising efficacy—Kim et al. showed, in a murine orthotopic GL261 glioma model, that the addition of anti-TIM-3 therapy to stereotactic radiosurgery (SRS), as well as triple therapy with anti-TIM3, anti-PD-1, and stereotactic radiosurgery, significantly increases overall survival and therefore needs to be studied in detail for clinical implementation. Compared to untreated mice with OS at 22 days, the addition of anti-TIM3 alone did not show a significant difference, while stereotactic radiosurgery vs. SRS + anti-TIM-3 antibody significantly increased median survival from 27 to 100 days; triple therapy with anti-PD-1 and anti-TIM-3 antibody and SRS showed 100% overall survival rate compared to SRS alone [279].

MBG-453 is an antibody against TIM3 in an ongoing phase I trial (NCT03961971). That clinical trial is the only one available on Clinicaltrials.gov with the query “Glioma|TIM-3” relevant for glioma treatment; at the time of writing of the manuscript for this review (May 2023), the trial was in the active, not recruiting, stage, with no results available.

#### 5.2.4. LAG-3

LAG-3 is a marker of active immune cells [280,281] with a similar mode of immune evasion in cancers such as PD-1 [282]. In tumors, LAG-3 is usually found within T cells that have lost their functions and, therefore, they could be a potential target for immune checkpoint inhibitors in oncology treatment [283,284].

In GBM, the LAG-3 expression profile correlates with the expression of the CD8+ T-cells CD8A [285]; therefore, targeting LAG-3 in glioblastomas with high levels of CD8+ cells in the tumor environment might be a beneficial clinical strategy. Currently, two phases I studies for the anti-LAG-3 monoclonal antibody are in the process of investigation (NCT02658981 and NCT03493932).

### 5.3. Targeting Extracellular Matrix (ECM) Components in Glioma Microenvironment

The extracellular matrix in brain tumors is a promising target for therapeutic treatment, as components of the brain ECM in pathologic conditions show tumor-specific behavior and ECM-dependent metabolic activity [286], which makes it possible to differentiate between targeting healthy tissue and the tumor environment in therapeutic development. The extracellular matrix in the brain tumor environment overexpresses several markers that are possible targets for glioma-specific therapies, such as tenascin-C, fibulin-3, fibronectin, and hyaluronan; as well as this it shows increased activity of metalloproteinase MMP9 [287].

Preclinical trials of the tenascin-C A1-targeting scFv IgE based antibody showed good accumulation in a tumor site in xenograft mice bearing U87 glioma cells, with comparable results to fibronectin-targeting antibody that is undergoing clinical trials at the moment [288].

A novel murine fibulin-3-targeting monoclonal antibody, mAB428.2, inhibited tumor growth in vivo in a xenograft model using GBM34 cells, as well as showing a pro-inflammatory effect and M2-macrophage infiltration into the tumor site [278]. Additionally, this study also tested the in vivo efficacy for intracranially placed fibulin-3-expressing tumor cell line GBM09 and showed increased median survival by 32% when the drug was delivered directly to the tumor area in the brain but not when administered systemically [289]. Further propagation of studies with fibulin-3-targeting drugs is needed for them to reach clinical trials.

Fibronectin is a promising target for delivering drugs to the extracellular compartment of solid tumors as it is a good marker of neovascularization and therefore could be targeted for eliminating the spread of the tumor tissue [290]. Preliminary in vivo studies showed that a radiolabeled scFv fragment of the L19 antibody that targets an ED-B domain of fibronectin significantly prolongs survival in mice with intracranially placed C6 glioma cells (22 vs. 16 days in mice bearing tumors of which the volume exceeded 150 mm^3^ [291]), as well as it showing preferential binding in the neovasculature region of the tumor, which is increased when antivasculature drugs are coadministered [290].

A modified herpes simplex virus bearing the sequence of the *MMP9* gene and EGFR/EGFRvIII-specific scFv for glioblastoma targeting was tested in a preclinical study within a murine model with implanted GBM30 cells. Results from this study showed that glioma-targeted MMP-9 virotherapy increased survival from 21 days in the control group treated with PBS compared to more than 60 days in the virotherapy group [292]. However, investigators were not sure if the applied treatment was responsible for a decrease in tumor volume due to the addition of the *MMP9* in the viral vector, as there was no statistical difference between the groups tested with the *MMP9*-armed and unarmed viruses [292].

## 6. Future Prospects and Conclusions

Gliomas, as representatives of CNS tumors, are characterized by a vast variety of altered markers within tumor cells and the surrounding microenvironment [2], with glioblastoma diagnosis holding poor survival rates and prognosis [1,3,4]. Many efforts in preclinical and clinical research are being invested into developing anti-glioma and GBM drugs; however, in many cases, these attempts are proving to be ineffective due to factors such as tumor location in the brain that is protected from main circulation due to presence of the BBB [293], as well as inter- and intratumoral heterogeneity [5,6,294,295,296], often resulting in acquired therapy resistance. Nonetheless, accumulating data on unsuccessful trials plays a great role in enlarging the understanding of gliomas from a fundamental science point of view and helps to elucidate the mechanisms of drug resistance for future improvements in therapeutic strategies.

Analysis of the current state of clinical approaches towards glioma treatment favors combinatorial treatment rather than a nanotherapeutic one in attempts to prolong survival and increase the quality of life for patients diagnosed with GBM, which goes by recent publications reviewing single-agent drugs versus a combination of treatments [297,298]. In preparation for the review, we observed that the majority of clinical trials with available results regarding glioma treatment do not show statistical significance in comparative studies or do not present statistical data at all. We observed that one study utilizing anti-EGFR-targeted antibody combined with standard-of-care radiotherapy + temozolomide (NCT02573324), and nine studies using bevacizumab in combinatorial treatment [32,220,221,222,223,224,225,226,266], showed statistically significant improvements in patients’ progression-free survival rate with no effect on OS, and one study involving anti PD-1 monoclonal antibody administered as adjuvant vs. neoadjuvant with concomitant surgery [265] showed an increase in both PFS and OS.

Novel approaches, such as photodynamic therapy [153,155,156,157] and utilization of oncolytic viruses [162], showed a significant increase in patients’ overall survival rates in clinical studies as compared with the standard-of-care Stupp protocol. Investigation of novel drugs as well as different delivery methods should be preferential in order to develop efficient therapy in GBM.

As outlined and cited within this review, the body of research for glioma treatment is an ever-growing field that is being developed rapidly as clinical scientists try to overcome the obstacles of inefficient treatment and unwanted toxicity within a tight timeframe before the patient succumbs to the pathology. The growing body of data regarding in vivo research that tests novel targeting strategies creates a potential pool for opportunities for future clinical trials and therefore clinical application.

However, patient stratification and study design play an important role in evaluating the efficiency of treatment. Antiangiogenic treatment, specific EGFR-targeting, and immune-checkpoint blockade could be a possible line of further improvement in existing treatments and investigational drugs, though it is important to note that the comparison and evaluation of results in glioma clinical research could be complicated due to very low therapeutic success and a low cohort of diagnosed GBM patients entering clinical trials (<5%) [299]. As a result of that, clinical research could use historical controls that might be unrelated to the current standard-of-care for therapy, which in turn can produce unreliable results [19,299,300]. Failure in the predictive potential of Phase II towards Phase III clinical trials in glioblastoma necessitates the reformatting of clinical trial development, with special attention towards cohorts of reference in comparative studies [300]. An additional obstacle within the correct evaluation of survival and drug efficacy could be due to the fact that some participants are enrolled in clinical trials only when the other treatment options have already been exhausted, creating variability in the survival analysis as different patients might have different treatment histories. Careful implementation of the growing body of knowledge regarding glioblastoma biology and response to treatment, combined with an improved study design, should be a point of preference for future studies on the matter, in order to develop targeted drugs that might improve patients’ PFS and OS.

## Figures and Tables

**Table 1:** Alterations in RTK expression within GBM, classified by the target RTK families used in GBM treatment.

Altered RTK	Occurrence in GBM and Type of Alteration
EGFR	A total of 57% of GBM cases show mutation, rearrangement, alternatively spliced isoforms or amplification [6]; around 20% of cases show mutant variant *EGFRvIII* [92]
PDGFR	A total of 13% of GBM harbor *PDGFRA* amplification or point mutations [6,92], *PDGFRB* showed overexpression on GBM endothelial cells [166]
VEGFR	*VEGF* is present in 64% of GBM [167] with 6–17% accounting for *VEGFR2* amplification [168]
c-MET	A total of 1.6–13.1% of GBM cases harbor *c-MET* overexpression [6,169]; in around 4% of GBM, *c-MET* amplification is present [92,170]
FGFR	A total of 3.2% of GBM has amplification or point mutation in *FGFR2* and *FGFR3* [6]

**Table 2:** Clinical trials investigating EGFR-specific targeted drugs in glioma (full composition according to the general scheme of a delivery system).

Trial Code (Study Period)	Recognition Moiety	Linker	Payload	Type of Intervention (Outcome)
**Depatuximab–mafodotin**				
NCT02573324 (December 2015–April 2022)	anti-EGFR/vIII mAb	maleimidocaproyl	MMAF	Combined with radiotherapy and temozolomide (RT/TMZ) vs placebo + RT/TMZ (N = 619, PFS = 8.0 vs. 6.3 months (*p* = 0.029), OS = 18.9 vs. 18.7 months (*p* = 0.633))
**AMG-595**				
NCT01475006 (February 2012–April 2016)	anti-EGFRvIII mAb	maleimidocaproyl	DM1	Monotherapy (no survival data available, [179])
**D2C7-IT**				
NCT04547777 (July 2021–December 2025)	Bispecific anti-EGFRvIII/CD3 Ab	PCR fused	Pseudomonas Exotoxin A	Combined with anti-CD40 mAb (no survival data available)
NCT05734560 (February 2023–February 2028)	Bispecific anti-EGFRvIII/CD3 Ab	PCR fused	Pseudomonas Exotoxin A	Combined with anti-CD40 mAb (no survival data available)
**EGFR(V)-EDV-Dox**				
NCT02766699 (October 2016–June 2020)	Panitumumab scFv (anti-EGFR)	Anti-O-polysaccharide antibody to the component of the drug delivery system (Minicell) + G4S linker	doxorubicin	Monotherapy with pretreatment (N = 14, PFS = 1.6 months, OS = 9.7 months, [180])
**C225-ILs-dox**				
NCT03603379 (November 2018–November 2020)	Cetuximab Fab (anti-EGFR)	Maleimide groups	doxorubicin	Monotherapy (N = 9, PFS = 1.5 months, OS = 8 months, [181])

**Table 3:** Selected clinical trials investigating EGFR-specific targeted drugs in glioma (combined recognition moiety and a payload).

Trial Code (Study Period)	Recognition Moiety/Payload	Type of Intervention (Outcome)
**RO7428731**		
NCT05187624 (April 2022–February 2025)	bispecific anti-EGFRvIII/CD3 Ab	Monotherapy (no survival data available)
**AMG-596**		
NCT03296696 (April 2018–August 2021)	bispecific anti-EGFRvIII/CD3 Ab	Monotherapy (no survival data available, [188])
**Cetuximab**		
NCT00311857 (February 2006–September 2006)	anti-EGFR mAb	Combined with RT/TMZ (N = 17, PFS6 = 81% PFS12 = 37%, OS12 = 87% [189])
NCT00463073 (August 2006–December 2008)	anti-EGFR mAb	Combined with bevacizumab and irinotecan (N = 43, PFS = 16 weeks, OS = 30 weeks, [190])
**Panitumumab**		
NCT01017653 (February 2010–October 2011)	anti-EGFR mAb	Combined with irinotecan (N = 16, PFS6 = 12.5%, OS = 4.6 months; study terminated due not reaching the benchmark efficacy rule)
**Nimotuzumab**		
NCT00600054 (October 2007–December 2010)	anti-EGFR mAb	Combined with RT/TMZ vs. RT/TMZ (insignificant data on PFS/OS in experimental vs. control groups)
**GC1118**		
NCT03618667 (April 2018–July 2021)	anti-EGFR mAb	Monotherapy (no survival data available)
**Sym004**		
NCT02540161 (February 2016–April 2020)	mixture of two anti-EGFR mAbs	Monotherapy (N = 43, PFS: non-bevacizumab failures (18 mg/kg) = 1.81 months, bevacizumab failures (18 mg/kg) = 3.91 months, non-bevacizumab failures (24 mg/kg) = 3.55 months, bevacizumab failures (24 mg/kg) = 2.00 months; OS: non-bevacizumab failures (18 mg/kg) = 7.54 months, bevacizumab failures (18 mg/kg) = 5.51 months, non-bevacizumab failures (24 mg/kg) = 9.95 months, bevacizumab failures (24 mg/kg) = 5.39 months)
**Erlotinib**		
NCT00671970 (February 2007–April 2010)	EGFR-specific TKI	Combined with bevacizumab (N (Anaplastic glioma) = 32, PFS = 23.4 weeks, OS = 71.3 weeks; N (GBM) = 25, PFS = 18 weeks, OS = 44.6 weeks [191])
NCT00124657 (March 2005–September 2014)	EGFR-specific TKI	Monotherapy (N = 8 (Phase I, anaplastic astrocytoma), 1-year PFS = 0.75 years; N = 12 (Phase I, GBM), 1-year PFS = 0.33 years; N = 20 (Phase II, anaplastic astrocytoma), 1-year PFS = 0.45 years, 2-year PFS 24 = 0.15 years; N = 21 (Phase II, GBM), 1-year PFS = 0.19 years, 2-year PFS = 0.19 years)
NCT00672243 (April 2007–December 2009)	EGFR-specific TKI	Combined with sirolimus (N = 32, PFS = 6.9 weeks, OS = 33.8 weeks [192])
NCT00112736 (April 2005–April 2014)	EGFR-specific TKI	Combined with temsirolimus (N = 16 (Anaplastic glioma), PFS6 = 8%; N = 42 (GBM), PFS6 = 13% [193])
NCT00301418 (March 2006–May 2014)	EGFR-specific TKI	Monotherapy (N = 11, PFS = 1.9 months, OS = 6.9 months [194])
NCT00720356 (July 2009–July 2018)	EGFR-specific TKI	Combined with bevacizumab (N = 46, OS = 13.2 months)
NCT00187486 (August 2004–March 2011)	EGFR-specific TKI	Combined with RT/TMZ (N = 65, PFS = 8.2 months, OS = 19.3 months [195])
NCT00445588 (January 2007–August 2009)	EGFR-specific TKI	Combined with sorafenib (N = 56, PFS = 2.5 months, OS = 5.7 months [196])
NCT00086879 (May 2004–March 2011)	EGFR-specific TKI	Monotherapy vs. TMZ or monotherapy vs. carmustine (N = 54, PFS6 (erlotinib arm) = 11.4%; N = 56, PFS6 (control arm) = 24% [197])
NCT00525525 (September 2007–May 2013)	EGFR-specific TKI	Combined with bevacizumab + TMZ in adjuvant therapy (N = 59, PFS = 13.5 months, OS = 19.8 months [198])
**Gefitinib**		
NCT01310855 (May 2011–January 2014)	EGFR-specific TKI	cediranib + gefitinib vs. cediranib + placebo (N = 97, PFS = 3.6 vs. 2.8 months, OS = 7.2 vs. 5.5 months [199])
NCT00250887 (July 2005–May 2007)	EGFR-specific TKI	Monotherapy (N = 22, OS = 8.8 months [200])
**ERAS-801**		
NCT05222802 (February 2022–September 2025)	EGFR-specific TKI	Monotherapy (no survival data available)
**BDTX-1535**		
NCT05256290 (February 2022–March 2025)	Mutant-selective EGFR TKI	Monotherapy vs. combined with TMZ (no survival data available)

**Table 4:** Selected clinical trials investigating PDGFR-specific targeted drugs in glioma (combined recognition moiety and a payload).

Trial Code (Study Period)	Recognition Moiety/Payload	Type of Intervention (Outcome)
**MEDI-575**		
NCT01268566 (December 2010–April 2017)	anti-PDGFRα mAb	Monotherapy (N = 56, PFS = 1.4 months, OS = 9.7 months, [213])
**Olaratumab**		
NCT00895180 (May 2009–December 2017)	anti-PDGFRα mAb	Monotherapy (N = 40, PFS6 = 7.5%, OS = 34.3 weeks)

**Table 5:** Selected clinical trials investigating VEGF/VEGFR-specific targeted drugs in glioma (combined recognition moiety and a payload).

Trial Code (Study Period)	Recognition Moiety/Payload	Type of Intervention (Outcome)
**Bevacizumab**		
NCT01860638 (May 2013–April 2018)	anti-VEGF-A mAb	Treatment with the Stupp protocol, combined with lomustine in the first progression and with chemotherapy in the second progression (N = 296, PFS (first progression) = 2.3 vs. 1.8 months, OS (first progression) = 6.4 vs. 5.5 months (bevacizumab + lomustine vs. lomustine + placebo), PFS (second progression) = 2.0 vs. 2.2 months (bevacizumab + chemotherapy vs. chemotherapy), [220])
NCT01443676 (September 2011–November 2016)	anti-VEGF-A mAb	Combined with RT vs. RT alone (N = 75, PFS = 7.6 vs. 4.8 months, OS = 12.1 vs. 12.2 months, (bevacizumab + RT vs. RT), [221])
NCT00943826 (July 2009–September 2017)	anti-VEGF-A mAb	Combined with RT/TMZ vs. RT/TMZ (N = 921, PFS = 10.6 vs. 6.2 months, OS = 16.8 vs. 16.7 months (bevacizumab + RT/TMZ vs. RT/TMZ), [222])
NCT00345163 (June 2006–May 2017)	anti-VEGF-A mAb	Monotherapy vs. combined with irinotecan (N = 167, PFS = 4.2 vs. 5.6 months, OS = 9.2 vs. 8.7 months, PFS6 = 42.6% vs. 50.3% (bevacizumab vs. bevacizumab + irinotecan), [223])
NCT01730950 (November 2012–December 2022)	anti-VEGF-A mAb	Monotherapy vs. combined with RT (N = 182, PFS = 7.1 vs. 3.8 months, OS = 10.1 vs. 9.7 months [224])
NCT00967330 (August 2009–November 2015)	anti-VEGF-A mAb	Combined with RT, then adjuvant therapy combined with irinotecan vs. RT/TMZ (N = 182, PFS = 5.99 vs. 9.7 months, OS = 16.6 vs. 17.5 months (RT/TMZ vs. RT + bevacizumab and irinotecan, [225])
NCT01290939 (February 2011–February 2021)	anti-VEGF-A mAb	Monotherapy vs. combined with lomustine (N = 437, PFS = 1.5 vs. 4.2 months, OS = 8.6 vs. 9.1 months; (bevacizumab vs. combined with lomustine, [226])
NCT00884741 (April 2009–July 2019)	anti-VEGF-A mAb	Combined with TMZ as adjuvant therapy vs. RT/TMZ (N = 637, PFS = 10.7 vs. 7.3 months, OS = 15.7 vs. 16.1 months, (bevacizumab + RT/TMZ vs. RT/TMZ, [32])
**CT-322**		
NCT00562419 (November 2007–October 2010)	VEGFR-2 specific extracellular modified fibronectin 10th type 3	Monotherapy vs. combined with irinotecan (N = 63; Monotherapy cohort: PFS6 (1 mg/kg) = 18.6%, PFS6 (2 mg/kg) = 0.0%; Combined with irinotecan: PFS6 (1 mg/kg) = 64.3%, PFS6 (2 mg/kg) = 42.1%, [227])

**Table 6:** Selected clinical trials investigating c-MET or HGF-targeted drugs in glioma (combined recognition moiety and a payload).

Trial Code (Study Period)	Recognition Moiety/Payload	Type of Intervention (Outcome)
**Onartuzumab**		
NCT01632228 (July 2012–February 2018)	anti-cMET mAb	Combined with bevacizumab vs. bevacizumab + placebo (N = 64; PFS = 3.9 vs. 2.9 months (*p* = 0.7774), OS = 8.8 vs. 12.6 months (*p* = 0.1389); (combined with bevacizumab vs. bevacizumab + placebo), [236])
**Rilotumumab**		
NCT01113398 (April 2010–December 2015)	anti-HGF mAb	Combined with bevacizumab, (N = 60; PFS = 4.1 vs. 4.3 weeks, OS = 6.5 vs. 5.4 months (10 vs. 20 mg/kg cohorts), [237])

**Table 7:** A clinical trial investigating FGFR-targeted drug in glioma (combined recognition moiety and a payload).

Trial Code (Study Period)	Recognition Moiety/Payload	Type of Intervention (Outcome)
**Infigratinib**		
NCT01975701 (November 2013–December 2019)	pan-FGFR TKI	Monotherapy (N = 26; PFS = 1.7 months, OS = 6.7 months)

**Table 8:** Selected clinical trials investigating multikinase inhibitors in glioma (combined recognition moiety and a payload).

Trial Code (Study Period)	Recognition Moiety/Payload	Type of Intervention (Outcome)
**Imatinib**		
NCT00290771 (February 2006–May 2011)	PDGFRα/β, BCR-Abl, c-Kit multitarget TKI	Combined with hydroxyurea (N = 231, PFS = 5.6 weeks, OS = 26 weeks [248])
NCT00154375 (September 2005–April 2011)	PDGFRα/β, BCR-Abl, c-Kit multitarget TKI	Combined with hydroxyurea vs. only hydroxyurea (N = 240, PFS = 6 vs. 6 weeks (imatinib + hydroxyurea vs. hydroxyurea alone); OS = 21 vs. 19 weeks (imatinib + hydroxyurea vs. hydroxyurea alone) [249])
NCT00039364 (January 2003–July 2012)	PDGFRα/β, BCR-Abl, c-Kit multitarget TKI	Monotherapy (N = 50 (GBM), PFS = 1.8 months, OS = 5.9 months; N = 25 (anaplastic astrocytoma), PFS = 1.8 months, OS = 5.0 months; N = 35 (oligodenrdroglioma), PFS = 1.9 months, OS = 5.3 months [250])
**Dasatinib**		
NCT00892177 (May 2009–October 2019)	PDGFRβ, EPHA2, BCR-Abl, c-Kit and SRC multitarget TKI	Combined with bevacizumab vs. bevacizumab alone (N = 121, PFS6 = 28.9% vs. 18.4% for dasatinib/bevacizumab vs. bevacizumab alone, no statistical significance; OS = 7.3 vs. 7.7 months for dasatinib/bevacizumab vs. bevacizumab alone, no statistical significance [251])
NCT00423735 (January 2007–July 2019)	PDGFRβ, EPHA2, BCR-Abl, c-Kit and SRC multitarget TKI	Monotherapy (N = 50, PFS = 1.7 months, OS = 7.96 months [252])
NCT00948389 (July 2009–August 2012)	PDGFRβ, EPHA2, BCR-Abl, c-Kit and SRC multitarget TKI	Combined with lomustine (N = 26, PFS = 1.35 months, OS = 6.4 months [253])
NCT00869401 (March 2009–February 2020)	PDGFRβ, EPHA2, BCR-Abl, c-Kit and SRC multitarget TKI	Combined with RT/TMZ vs. RT/TMZ (N = 204, PFS: 15.6 vs. 19.3 months; OS = 6.2 vs. 7.8 months, no statistical significance)
**Tandutinib**		
NCT00379080 (September 2009–April 2017)	PDGFRβ, FLT3, c-Kit multitarget TKI	Monotherapy (N = 31, PFS = 1.9 months, OS = 8.8 months [254])
NCT00667394 (April 2008–November 2015)	PDGFRβ, FLT3, c-Kit multitarget TKI	Combined with bevacizumab (N = 41, PFS = 4.1 months, OS = 11 months [255])
**Sunitinib**		
NCT01100177 (April 2010–March 2013)	PDGFRα/β, c-Kit, VEGFR1/2/3, FLT3 and RET multitarget TKI	Monotherapy prior, during and after RT (N = 12, PFS = 7.7 weeks, OS = 12.8 weeks [256])
NCT00923117 (June 2009–September 2015)	PDGFRα/β, c-Kit, VEGFR1/2/3, FLT3 and RET multitarget TKI	Monotherapy in patients with/without resistance to bevacizumab (N = 87, PFS (bevacizumab resistant cohort) = 0.92 months, PFS (bevacizumab naïve cohort) = 1.08 months)
NCT00535379 (September 2007–August 2010)	PDGFRα/β, c-Kit, VEGFR1/2/3, FLT3 and RET multitarget TKI	Monotherapy (N = 70, PFS = 2.2 months, OS = 9.2 months [257])
NCT00606008 (February 2008–November 2012)	PDGFRα/β, c-Kit, VEGFR1/2/3, FLT3 and RET multitarget TKI	Monotherapy (N = 16 (GBM), PFS = 1.4 months, OS = 12.6 months; N = 14 (AA), PFS = 4.1 months, OS = 12.1 months [258])
NCT00499473 (July 2007–February 2016)	PDGFRα/β, c-Kit, VEGFR1/2/3, FLT3 and RET multitarget TKI	Monotherapy in patients taking/not taking enzyme induced Anticonvulsants (EIAC) (N = 27 (non-EIAC), OS = 5.7 months; N = 4 (EIAC), OS = 12.3 months)
**Ponatinib**		
NCT02478164 (June 2015–July 2018)	BCR-Abl, PDGFRα, VEGFR2, FGFR1, and Src multitarget TKI	Monotherapy (N = 15, PFS = 28 days, OS = 98 days [259])
**Cediranib**		
NCT01062425 (February 2010–May 2022)	VEGFR, c-Kit, PDGFR multitarget TKI	Combined with RT/TMZ vs. RT/TMZ (N = 261; PFS = 6.2 vs. 2.7 months (*p* = 0.03), OS = 14.5 vs. 13.8 months (*p* = 0.44)(Cediranib + RT/TMZ vs. RT/TMZ))

**Table 9:** Selected clinical trials investigating anti-PD1/PD-L1-targeted drugs in glioma (combined recognition moiety and a payload).

Trial Code (Study Period)	Recognition Moiety/Payload	Type of Intervention (Outcome)
**Pembrolizumab**		
NCT02337686 (January 2015–January 2023)	anti-PD-1 mAb	Combined with surgery (N = 18, PFS = 2.4 months vs. 3.3 months (*p* = 0.03), OS = 7.5 months vs. 13.7 months (*p* = 0.04) for adjuvant vs. neoadjuvant application [265])
NCT02337491 (January 2015–December 2020)	anti-PD-1 mAb	Monotherapy vs. combined with bevacizumab (N = 80; PFS = 1.4 vs. 4.1 months (*p* = 0.0026), OS = 10.3 vs. 8.8 months (*p* = 0.87) (monotherapy vs. combined with bevacizumab) [266])
NCT02054806 (February 2014–May 2021)	anti-PD-1 mAb	Monotherapy vs. combined with bevacizumab (N = 26, PFS = 2.8 months, OS = 13.1 months [267])
**Nivolumab**		
NCT02017717 (December 2013–June 2022)	anti-PD-1 mAb	Monotherapy vs. bevacizumab (N = 369; PFS = 1.5 vs. 3.5 months, OS = 9.8 vs. 10.0 months, (nivolumab vs. bevacizumab) [268])
NCT02667587 (January 2016–April 2023)	anti-PD-1 mAb	Combined with RT/TMZ vs. RT/TMZ + placebo (N = 716; PFS = 10.6 vs. 10.3 months, OS = 28.9 vs. 32.1 months, (nivolumab + RT/TMZ vs. RT/TMZ + placebo) [269])
NCT02617589 (December 2015–March 2023)	anti-PD-1 mAb	Combined with RT vs. RT/TMZ (N = 560; PFS = 6.0 vs. 6.2 months, OS = 13.4 vs. 14.9 months, (nivolumab + RT vs. RT/TMZ) [270])
**Durvalumab**		
NCT02336165 (January 2015–October 2022)	anti-PD-L1 mAb	Cohort A (newly diagnosed): Combined with RT, Cohort B (bevacizumab-naïve): Monotherapy; Cohort B2 (bevacizumab-naïve): Combined with 10 mg/kg bevacizumab; Cohort B3 (bevacizumab-naïve): Combined with 3 mg/kg bevacizumab, Cohort C (bevacizumab-refractory): Combined with 10 mg/kg bevacizumab (N (A) = 40, N (B) = 31, N (B2) = 33, N (B3) = 33, N (C) = 22; PFS: A = 4.6, B = 3.0, B2 = 3.7, B3 = 3.7, C = 1.9 months, OS: A = 15.1, B = 6.7, B2 = 8.7, B3 = 9.3, C = 4.5 months [271])

**Table 10:** A clinical trial investigating CTLA-4-targeted drug in glioma (combined recognition moiety and a payload).

Trial Code (Study Period)	Recognition Moiety/Payload	Type of Intervention (Outcome)
**Tremelimumab**		
NCT02794883 (June 2016–April 2022)	Anti-CTLA-4 mAb	Monotherapy (A) vs. durvalumab (B) vs. combined with druvalumab (C) (N = 36; PFS: A = 2.746, B = 4.356, C = 4.913 months; OS: A = 7.246, B = 11.71, C = 7.703 months)

## Abbreviations

BBB	Blood–brain barrier
CNS	Central Nervous System
CTLA-4	Cytotoxic T-Lymphocyte-Associated Protein 4
DM1	mertansine
DM4	ravtansine
DSPE-PEG	1,2-distearoyl-sn-glycero-3-phosphoethanolamine-N-[amino(polyethylene glycol)]
ECM	Extracellular Matrix
EGFR	Epidermal Growth Factor Receptor
EPR	Enhanced Permeation and Retention
FDA	Food and Drug Administration
FGFR	Fibroblast Growth Factor Receptor
GBM	glioblastoma multiforme
HGF	Hepatocyte Growth Factor
HGG	High-Grade Gliomas
LGG	Low-Grade Gliomas
MDR	Multidrug Resistance
MET/c-MET	Mesenchymal–Epithelial Transition factor
MMAE	Monomethyl Auristatin E
MMAF	Monomethyl Auristatin F
MRP	MDR-Associated Protein
OS	Overall Survival
OS12	12-month OS
PBS	Phosphate-Buffered Saline
PD-1	Programmed Cell Death Protein 1
PDGFR	Platelet-Derived Growth Factor Receptor
PD-L1	Programmed Cell Death Ligand 1
PDT	Photodynamic Therapy
PFS	Progression-Free Survival
PFS12	12-month PFS
PFS6	6-month PFS
RT	Radiotherapy
RTK	Receptor Tyrosine Kinase
SELEX	Systematic Evolution of Ligands by Exponential Enrichment
SRS	Stereotactic Radiosurgery
TKI	Tyrosine Kinase Inhibitor
TMZ	Temozolomide
VEGF	Vascular Endothelial Growth Factor
VEGFR	Vascular Endothelial Growth Factor Receptor
WHO	World Health Organization
